# Antibiotic susceptibility pattern of Campylobacter sp. isolated from human stool samples including comparison of ellipsoid test and broth microdilution for meropenem

**DOI:** 10.3205/id000092

**Published:** 2025-06-17

**Authors:** Juliane Fornefett, Sangeeta Banerji, Dagmar Rimek

**Affiliations:** 1 Thuringian State Authority of Consumer Protection, Department of Health Protection, Bad Langensalza, Germany; 2 Robert Koch Institute, National Reference Center for Salmonella and Other Enteric Pathogens, Wernigerode, Germany

**Keywords:** Campylobacter, meropenem, resistance, susceptibility testing

## Abstract

Foodborne campylobacteriosis is the most common cause of bacterial gastroenteritis in Germany. Due to increasing antibiotic resistance in *Campylobacter*, data of isolates of human origin are published by the European Center for Disease Control and Prevention (ECDC)/European Food Safety Authority (EFSA). However, data on susceptibility to meropenem, an antibiotic of last resort, is not included. Therefore, the minimal inhibitory concentration (MIC) for meropenem was measured in 125 *Campylobacter* *jejuni* (*Cj*) and 57 *Campylobacter* *coli* (*Cc*) isolates isolated from human stool samples between 2020 and 2023, comparing ellipsoid test and broth microdilution.

Additionally, we determined the susceptibility of 249 *Cj* and 84 *Cc* strains isolated between 2018 and 2023 to erythromycin, ciprofloxacin and oxytetracycline by disk diffusion according to the European Committee on Antimicrobial Susceptibility Testing (EUCAST).

For meropenem, the MIC results of 5% *Campylobacter* isolates were interpreted as resistant. Erythromycin resistance was found in none *Cj* versus 9 (11%) *Cc* isolates that were resistant to all three substances. Ciprofloxacin and oxytetracycline resistance were detected in 72 and 41% *Cj*, and 67 and 70% *Cc* isolates, respectively. Only 24% *Cj* and 13% *Cc* isolates were susceptible to all three substances. The dual resistance of ciprofloxacin and oxytetracycline was the most common resistance pattern, observed in 37% *Cj* and 38% *Cc* isolates, respectively. None of the isolates was resistant to all four tested substances.

Our data underline the need for susceptibility testing of *Campylobacter* to alternatively used antimicrobial substances in clinical laboratories. The ellipsoid test provides a good alternative for meropenem MIC testing, although borderline isolates should be confirmed using microdilution.

## Introduction

*Campylobacter* (*C*.) species are the most common cause of bacterial gastroenteritis in Germany [1]. They are carried in the intestinal tract of animals and can contaminate food of animal origin during processing. Antibiotic treatment in humans is necessary for severe or prolonged infections only, but antibiotic-resistant *Campylobacter* strains are becoming an increasing problem. In this context, the German Federal Office of Consumer Protection and Food Safety (BVL) publishes data on prevalence and resistance of *Campylobacter* sp. of animal and food origin in its annual Zoonosis Monitoring Report [2], [3], [4], [5]. Epidemiological data on human infections in Germany are published by the Robert Koch Institute (RKI) in its Epidemiology Yearbook on Infectious Diseases [6]. However, information about resistance patterns are not included. In 2020, the RKI initiated a national surveillance program on *Campylobacter* to systematically collect epidemiological and resistance data from all over Germany. At the European level, the European Center for Disease Control and Prevention (ECDC) collaborates with the European Food Safety Authority (EFSA) to regularly publish prevalence and resistance data of zoonotic pathogens of animal, food, and human origin [7]. The European authorities receive and consolidate raw data from respective national reference centers (RKI and the German Federal Institute for Risk Assessment (BfR)) in Germany). 

The Thuringian State Authority for Consumer Protection (TLV) established a local resistance surveillance program for macrolides, quinolones, and tetracyclines in 2018. Since 2020, additional data on minimum inhibitory concentration (MIC) for meropenem were collected. This drug may be considered an alternative therapy for severe infections like meningitis, especially with multiresistant isolates [8], but surveillance data of susceptibility testing are not available.

Here we report the results of susceptibility testing for *Campylobacter* isolates gathered between 2018 and 2023 in Thuringia, Germany, with a focus on susceptibility to meropenem.

## Material and methods

### Collection of stool samples

This study was performed at the Thuringian State Authority for Consumer Protection (TLV) from 2018 to 2023. The laboratory of the TLV received stool samples of patients with diarrhea and contact persons from the local public health authorities in Thuringia for microbiological analysis. Since 2018, all samples that were examined for *Campylobacter* spp. were included in the study.

### Cultural detection and identification of Campylobacter species from stool samples

For cultivation of *Campylobacter* spp., human stool specimen were cultivated on blood free *Campylobacter* selective agar according to Karmali (Oxoid Deutschland GmbH, Wesel, Germany) in jars at 42°C for 48 h under microaerophilic conditions (Anoxomat Advanced Instruments, Norwood, MA, USA; 5.9% O2, 3.6% CO2, 7.2% H2, 83.3% N2). Species identification was performed from *Campylobacter*-suspected, oxidase-positive colonies using MALDI TOF mass spectrometry (Microflex LT instrument, Biotyper 3.1 software, Bruker Daltonik GmbH, Bremen, Germany).

### Meropenem testing using the ellipsoid test

For antimicrobial susceptibility testing of meropenem, a fresh overnight culture on Columbia Agar with sheep blood (Oxoid Deutschland GmbH, Wesel, Germany) was used that was grown at 42°C in jars under microaerophilic conditions, see above.

The determination of MIC values for meropenem (0.016 to 256 µg/ml) by ellipsoid test was performed using MIC test strips (Liofilchem Diagnotici, Roseto degli Abruzzi, Italy) on Mueller Hinton Agar with horse blood under identical conditions as described below for disc diffusion. Due to the absence of clinical breakpoints or epidemiological cut-off values (ECOFF), the EUCAST (European Committee on Antimicrobial Susceptibility Testing) ECOFF for *C. jejuni* and ertapenem (0.125 µg/ml [9]) was used in accordance with the recommendations of the German National Reference Laboratory for *Salmonella* and Other Bacterial Enteric Pathogens located at the RKI [internal communication]. Isolates with MICs at or below the ECOFF (‘wild-type’) were interpreted as susceptible. Isolates with MICs above the ECOFF (‘non-wild-type’) were interpreted as resistant.

### Meropenem testing using the broth micro dilution method

Susceptibility testing of meropenem using broth microdilution was performed at the National Reference Center for *Salmonella* and Other Enteric Pathogens placed at the RKI. 

This method was based on the EUCAST guidelines with some modifications in key aspects: a 24-hour liquid culture in *Brucella* broth (Thermo Fisher Scientific, Karlsruhe, Germany) incubated under microaerophilic conditions (5% O_2_) at 42°C was used. Hundred µl of this liquid culture were diluted 1:100 with a 0.85% saline solution. From this dilution, 10 µl were added to 100 µl of meropenem solution in *Brucella* broth in a concentration range of 0.016 to 8 µg/ml. After 24 to 48 h of incubation under microaerophilic conditions (5% O_2_) at 42°C the MIC was determined visually. MICs were interpreted as described above. This methodology had been validated through successful participation in an international proficiency testing program conducted by the Danish Statens Serum Institute (SSI Copenhagen) with the following antimicrobials: ciprofloxacin, erythromycin, and tetracycline.

### Performance criteria for the comparison of ellipsoid test method against broth micro- dilution method for determination of MICs for meropenem

Standard performance criteria included ≥90% essential and categorical agreement, ≤3% major errors, and ≤1.5% very major errors [10], [11]. 

The level of categorical agreement between the two methods was calculated as the percentage of isolates with the same interpretation (sensitive or resistant). To determine the level of agreement the Cohen’s kappa coefficient (r) was calculated. 

A susceptible result with the ellipsoid test and a resistant result by broth microdilution was defined as a very major error. A resistant result with the ellipsoid test and a susceptible result by broth microdilution was defined as a major error [12].

Essential agreement was defined as the percentage of isolates with a result being plus or minus one doubling dilution of that from broth microdilution [10], [11]. 

### Antimicrobial susceptibility testing for macrolides, quinolones, and tetracyclines by agar diffusion test

Antimicrobial susceptibility testing was performed routinely from all *Campylobacter* isolates. Erythromycin (ERY) was used as the reference substance for macrolides, ciprofloxacin (CIP) for quinolones, and oxytetracycline (OTC) for tetracyclines. For antimicrobial susceptibility testing, a fresh overnight culture on Columbia Agar with sheep blood (Oxoid Deutschland GmbH, Wesel, Germany) was used that was grown at 42°C in jars under microaerophilic conditions, see above.

Antimicrobial susceptibility testing for ciprofloxacin (CIP) (5 µg), oxytetracycline (OTC) (30 µg) and erythromycin (ERY) (15 µg) was performed on Mueller-Hinton agar plates with horse blood (Oxoid Deutschland GmbH, Wesel, Germany) using disc diffusion (Oxoid Deutschland GmbH, Wesel, Germany) according to the European Committee on Antimicrobial Susceptibility Testing (EUCAST). Zone diameters were read after 24 h of incubation at 42°C under microaerophilic conditions, and interpreted according to EUCAST Clinical Breakpoint Tables Versions 7.1 to 13.0 (years 2017 to 2023) [13]. Isolates with results that were interpreted as “Susceptible, increased exposure (I)” according to EUCAST Version 11.0 (2021) were classified as “Susceptible (S)” to ensure clarity in the statistics. *C. jejuni* ATCC 33560 was used as a quality control strain.

## Results

### Epidemiological background of the Campylobacter isolates under investigation

A total of 6.592 stool samples of different patients were examined for *Campylobacter* sp. between 2018 and 2023. 

**Figure 1:**
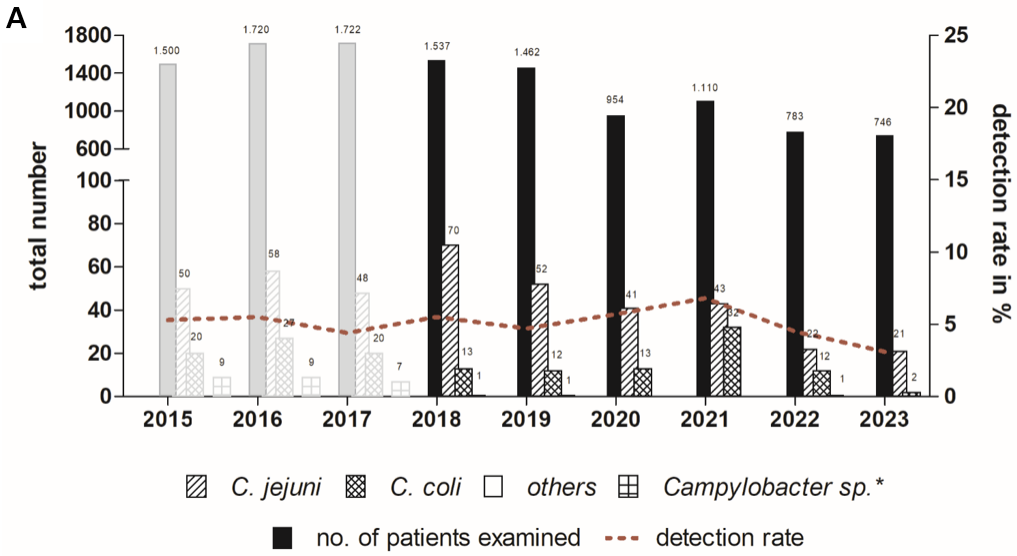
Numbers of stool samples and annual detection rates of *Campylobacter* spp. during the observation period 2018 to 2023 in comparison to previous years from the laboratory of the TLV * Species not specified

Overall, 5.1% (n=336) of these patients tested positive for *Campylobacter* sp. The yearly positivity rate varied between 3.1 and 6.8%, and corresponded to detection rates of the previous years 2015 to 2017 (Figure 1 (Fig. 1)). The high number of confirmed *C. jejuni* cases in 2018 was related to an outbreak in a kindergarten due to the consumption of inadequately heated raw milk. This outbreak was the largest in Germany that year. In 2022 and 2023, the number of samples and obtained isolates declined. Isolates originated from all Thuringian districts. 

A total of 249 isolates (74.1%) were identified as *C. jejuni*, 84 isolates (25%) as *C. coli*, 2 strains as *C. lanienae* (0.6%), and 1 isolate as *C. lari* (0.3%).

### Meropenem MIC values in C. jejuni and C. coli isolates by ellipsoid test

**Figure 2:**
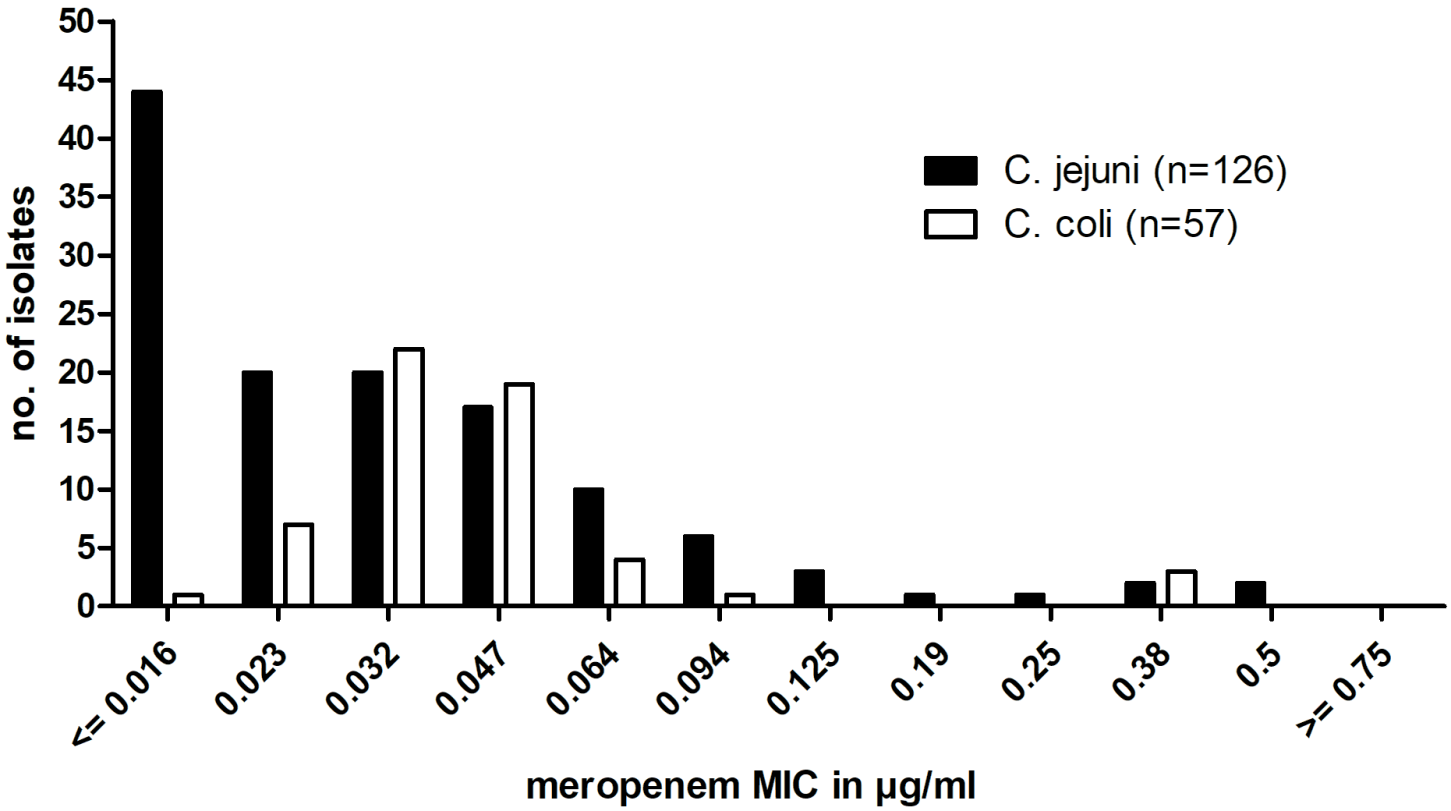
Meropenem MIC distribution in Thuringian *Campylobacter* *jejuni* and *Campylobacter* *coli* isolates between 2020 and 2023. MIC was determined using the ellipsoid test.

Between 2020 and 2023, the MICs for meropenem were determined in 126 *C. jejuni* and 57 *C. coli* isolates by the ellipsoid test. The MIC distribution of these results is shown in Figure 2 (Fig. 2). The MICs ranged from ≤0,016 to 0.5 µg/ml with 6 isolates (4.8%) of *C. jejuni* and 3 isolates (5.3%) of *C. coli* having an MIC above the chosen ECOFF of 0.125 µg/ml. These strains were therefore considered as resistant. The MIC_50_ for *C. coli* (0.032 µg/ml/l) was slightly higher than that for *C. jejuni* (0.023 µg/ml). 

Notably, none of the isolates was resistant to all 4 substances tested here.

### Comparison of MIC determination for meropenem using ellipsoid test and microdilution

Microdilution is the reference method for determining the minimum inhibitory concentration (MIC) of *Campylobacter* isolates. To evaluate the MIC determination for meropenem using agar diffusion (ellipsoid test) as a cost-effective and easy-to-perform method, we compared the results of both methods on 78 *C. jejuni* and 40 *C. coli* isolates. 

Results for categorical agreement, errors, Cohen’s Kappa coefficient and essential agreement are shown in Table 1 (Tab. 1) and Table 2 (Tab. 2). The level of categorical agreement for the interpretation as sensitive or resistant was 100% (r=1) with no major or very major errors. Essential agreement resulted in 82% and 85% for *C. jejuni* and *C. coli*, respectively (83% in total), with 14 *C. jejuni* and 6 *C. coli* isolates being tested with two or more dilution steps away from reference.

### Susceptibility patterns of Campylobacter isolates to macrolides, quinolones, and tetracyclines

**Figure 3:**
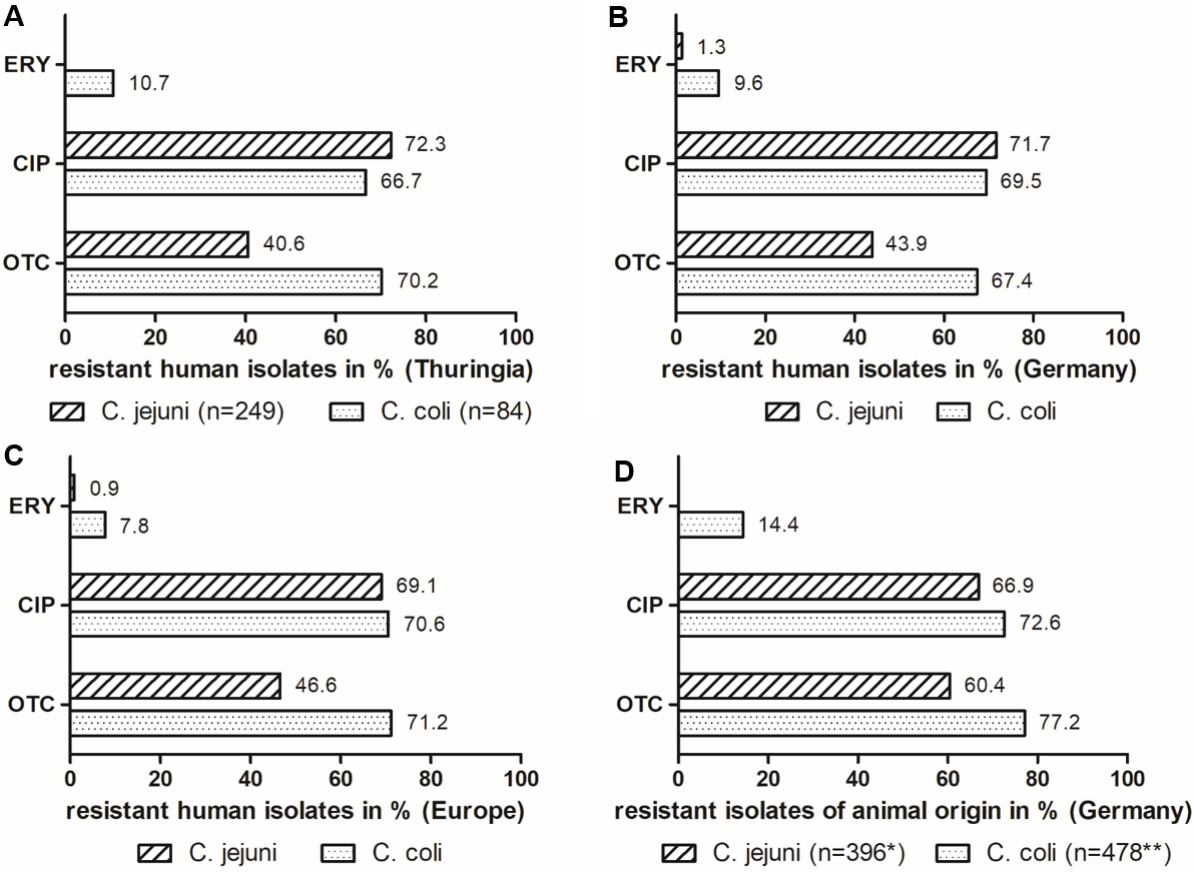
Resistances of *Campylobacter* *jejuni* and *Campylobacter* *coli* isolates to erythromycin (ERY), ciprofloxacin (CIP), and oxytetracyclin (OTC): (A) Thuringian human isolates collected between 2018 and 2023; (B) Resistance data of German human *Campylobacter* isolates adapted from the ECDC/EFSA “European Union Summary Report on Antimicrobial Resistance in zoonotic and indicator bacteria from humans, animals and food 2021 to 2022” [7]; (C) Resistance data of European human *Campylobacter* isolates adapted from the ECDC/EFSA Report [7]; (D) Combined resistance data of German isolates from animal origin adapted from the BVL Zoonoses Monitoring Reports 2021 and 2022 [2], [3] * Consisting of 120 broiler isolates, 140 turkey isolates, 3 pig isolates and 133 calve/heifer isolates ** Consisting of 31 broiler isolates, 148 turkey isolates, 258 pig isolates and 41 calve/heifer isolates

Results for *C. jejuni* and *C. coli* are shown in Figure 3A (Fig. 3) and Table 3 (Tab. 3). 

A total of 72 *Campylobacter* strains (21.4%), including 60 (24.1%) *C. jejuni*, 11 (13.1%) *C. coli* and 1 *C. lanienae*, were fully susceptible to macrolides, quinolones, and tetracyclines (Table 3 (Tab. 3)). As can be seen in Figure 3A (Fig. 3), resistance to erythromycin was rare and occurred in *C. coli* only (9 isolates, 10.7%). A total of 180 isolates (72.3%) of *C. jejuni*, 56 isolates (66.7%) of *C. coli*, 1 *C. lanienae* isolate, and 1 *C. lari* isolate were resistant to ciprofloxacin. Resistance to oxytetracycline was found in 101 isolates (40.6%) and 59 isolates (70.2%) of *C. jejuni* and *C. coli*, respectively.

Table 3 (Tab. 3) shows the distribution of single and combined resistance of* C. jejuni* and *C. coli* isolates to the three antibiotic substances: single resistance to erythromycin has not been detected so far. However, single resistance to ciprofloxacin was found in 88 (35.3%) and 14 (16.7%) isolates of *C. jejuni* and* C. coli*, respectively. Single resistance to oxytetraxycline was detected in 9 (3.6%) isolates of *C. jejuni* and 18 (21.4%) isolates of *C. coli*. 

The dual resistance of ciprofloxacin + tetracycline was the main detected resistance pattern and comprised 92 (36.9%) isolates of *C. jejuni* and 32 (38.1%) isolates of *C. coli*. Triple resistance was only found among *C. coli* isolates, with a total of 9 isolates (10.7%).

## Discussion

*Campylobacter* infections are self-limiting in most patients, but patients with severe or systemic disease require antibiotic treatment. Treatment options are limited due to intrinsic resistance of *Campylobacter* spp. to different antibiotic groups [14]. For the treatment of human campylobacteriosis, the previous German S2k guideline “Gastrointestinal Infections and Whipple’s Disease” from 2015 [15] recommended azithromycin as the first choice with ciprofloxacin as an alternative. Regarding the resistance data of previous years, this recommendation had long been outdated and urgently needed revision. This revision finally took place in November 2023. The current guideline recommends azithromycin [16]. Publications on other alternative therapy options, especially with carbapenems do exist [8], [14], [17], but they have not made their way into the recent German guideline. 

The resistance situation of *Campylobacter* spp. to the commonly used quinolones and tetracyclines in Thuringia is alarming, with resistance rates exceeding 67–72% and 41–70% (Figure 3A (Fig. 3)), respectively. On the other hand, the situation for *C. jejuni* and macrolides is still favorable, as we have not detected any resistant isolate so far. However, the trend for *C. coli* is already concerning with erythromycin-resistance rates of almost 11% (Figure 3A (Fig. 3)). It is also important to note that so far, erythromycin resistance always occurred in combination with ciprofloxacin and oxytetracycline resistance (Table 3 (Tab. 3)), which is a very critical combination. 

Data from Germany show a very similar picture (Figure 3B (Fig. 3)): according to the last European Union Summary report on Antimicrobial Resistance for 2021 to 2022 ([7], see Annex B: https://zenodo.org/records/10528846), resistance rates for erythromycin exceed 1–10%, for ciprofloxacin 70–72% and for tetracycline 44–67%. Resistance to the combination erythromycin + ciprofloxacin occurred at a rate of 1.2% for *C. jejuni* and 8.9% for *C. coli*.

The Thuringian data also compare well to the European average values, as reported in the European Union Summary Report [7]. However, some European member states have alarming high rates of resistance, whereas the situation in Thuringia appears to be moderate in comparison. The average resistance data from Europe adapted from the report are shown in Figure 3C (Fig. 3): 0.9 to 7.8% of *Campylobacter* isolates were resistant to erythromycin with higher values for *C. coli*. However, resistance rates especially for *C. coli* ranged from 0% (e.g. Austria, Estonia) to almost 39% (Greece). Peak values of more than 73% of erythromycin resistant C*. coli* were reported in previous years from Portugal [18]. The European resistance rate of ciprofloxacin and tetracycline ranges from 69 to 71% and 47 to 71%, respectively, with some member states reaching values above 93% for one or both antimicrobials (e.g. Portugal, Lithuania, Poland). In our study, the most common resistance pattern in *C. jejuni* and *C. coli* was the combined resistance against ciprofloxacin + oxytetracycline, which occurred in 37% and 38% of the isolates, respectively (Table 3 (Tab. 3)). This result corresponds to findings from other European countries [18], [19].

Furthermore, the critical resistance combination erythromycin + ciprofloxacin was reported in Europe for 0.7 and 7% of *C. jejuni* and *C. coli* isolates, respectively. Two member states (Portugal and Greece) even reported resistant *C. coli* isolates of up to 27%. In previous years, Portugal reported about 4% of its *C. jejuni* isolates and more than 70% of its *C. coli* isolates to be resistant [18]. In Thuringia, the erythromycin + ciprofloxacin resistance combination has not been detected in *C. jejuni* in this study. However, as described above, we detected this resistance pattern in almost 11% of *C. coli* isolates (Table 3 (Tab. 3)). 

Due to the inter-European and global movement of food, livestock, and people, it is obvious that clinicians in Germany will also be increasingly challenged with multidrug-resistant *Campylobacter* infections in the future. 

Even if only considering the data from Europe, it is not surprising that the World Health Organization (WHO) has classified *Campylobacter* sp. as a priority level 2 (high) pathogen on their Priority Pathogens List for R&D of new Antibiotics [20].

The β-lactam meropenem is another listed substance for alternative treatment of multidrug-resistant severe campylobacteriosis [17]. It has been successfully administered in systemic infections, but strains may acquire resistance during treatment [8], [21]. This reserve antibiotic is suitable due to its good tolerability, but it must be administered via intravenous injection. However, there are no clinical breakpoints or epidemiological cut-offs (ECOFF) for *Campylobacter* to meropenem. The use of ECOFFs from other substances or bacterial species can only be a temporary solution and remains unsatisfactory. Therefore, clinical studies on this topic are needed to supplement the EUCAST breakpoint tables in this regard. 

Our data confirm that it is justified to test *Campylobacter* isolates for resistance to common and alternative antibiotics in clinical laboratories, in order to address the alarming resistance situation in locally or internationally acquired campylobacterioses. Our findings suggest that for meropenem MIC testing, the ellipsoid test is a useful, cost-effective, and easy-to-perform alternative due to meeting the criteria for categorical agreement (≥90%) and major (≤3%) as well as very major errors (≤1.5%) (Table 1 (Tab. 1)). However, due to not meeting the criteria for essential agreement (<90%) (Table 2 (Tab. 2)), it is still recommended to confirm borderline isolates using the microdilution method. 

In Thuringia, the resistance rates for meropenem were low but not to be neglected at approximately 5% of *C. jejuni* and *C. coli* isolates (Figure 2 (Fig. 2)), so the use for treatment of severe multidrug resistant campylobacterioses can still be recommended. Notably, we found no isolates resistant to all four tested substances so far.

When dealing with human infections and resistances, it is also important to keep the current situation in food and food-producing animals as main infection sources in mind. Therefore, it is worth to consult the Zoonosis Monitoring Report published annually by the BVL [2], [3], [4], [5]. The predominant species in human infections are *C. jejuni* (>74%) and *C. coli* (>10%) [6]. Fifty to ninety percent of human *Campylobacter* cases are related to the consumption of chicken meat [1]. This is not surprising, considering that half of the fresh chicken meat in German retail is contaminated with *Campylobacter* species [2], [3], [4], [5]. The predominant species in chicken is *C. jejuni* [2], [3], [4], [5]. Turkey meat also serves as reservoir for human infections with contamination rates of 33% of samples in 2018 [4] and still 11% in 2022 [3]. Turkeys are colonized by *C. jejuni* and *C. coli* in almost equal measures [2], [3], [4], [5]. Pork and beef as well as milk is rarely affected, with a maximum of 1% of the samples contaminated (2015) [5]. However, pigs and cattle should also be attended, especially due to the consumption of raw minced meat, tartare and raw milk. Cattle is predominantly colonized by *C. jejuni*, while pigs are almost exclusively colonized by *C. coli* [2], [3], [4], [5]. 

Figure 3D (Fig. 3) provides an overview of the resistance patterns in *C. jejuni* and *C. coli* isolates isolated from livestock. In order to better compare the resistance rates of human and animal isolates, the data from different animal species, adapted from the zoonosis monitoring reports of the BVL [2], [3], is combined. This was done to ensure that the majority of potential livestock sources for human *Campylobacter* infections were considered, since we did not determine the infection sources for our patients. Furthermore, the resistance rates to erythromycin, ciprofloxacin, and tetracycline vary only moderately among *Campylobacter* isolates from different livestock species [2], [3]. The data of the BVL report include isolates from caecum samples of broiler chickens, turkeys, pigs, and calves/heifers that were collected in German slaughterhouses [2], [3]. Whether the animals originally came from Germany or abroad is not further explained in the report. In total, the data for *C. jejuni* and *C. coli* of animal origin compare well to the human data, showing low to moderate resistance rates to erythromycin (up to 14%) and high to very high resistance rates to ciprofloxacin and tetracycline (above 67% and 60%, respectively) (Figure 3D (Fig. 3)). The similar results of resistance monitoring in humans and animals highlight the necessity of the One Health approach in combating human campylobacteriosis. In this regard, special attention must be given to the prevention of transmission between livestock and humans. 

Despite adhering to all biosecurity measures in animal husbandry, eradication of *Campylobacter* from livestock is unrealistic. Measures to reduce *Campylobacter* load in livestock, such as the use of pre/probiotics, bacteriophages, bacteriocins, and vaccinations [22], or breeding *Campylobacter*-resistant livestock [23], are still experimental at this time. Measures to reduce contamination of carcasses, such as deep freezing/crust freezing and surface treatment using e.g. steam, ultrasound, radiation, or chemicals like lactic acid or chlorine [22], are also experimental or not approved in the EU. In 2018, the EU Regulation 2017/1495 introduced a process hygiene criterion at German slaughterhouses for the purpose of reducing and monitoring Campylobacter contamination on chicken carcasses. However, so far, we have observed only a slight decrease in human campylobacterioses in Germany [24] and Thuringia, which raises doubts about the effectiveness of the measure. Since an effective reduction of *Campylobacter* burden is probably not achievable in either animal husbandry or at the slaughterhouse level, prevention at the consumer level remains crucial. Thus, it is especially important to educate people about the pathogen, basic kitchen hygiene measures, and risks associated with handling and consuming raw food. 

## Conclusions

- The resistance situation of *Campylobacter* spp. for macrolides is currently favorable, while it is critical for quinolones and tetracyclines. Increasing macrolide and critical multidrug resistances are already being detected in isolates from European countries. Therefore, it is urgently necessary to validate alternative antibiotics for treatment and establish clinical breakpoints for susceptibility testing of these substances.
- The current data underline the necessity of systematic resistance testing of human *Campylobacter* isolates in clinical laboratory practice. The central collection and regular publication of resistance data is reasoned.
- In Thuringia, the resistance situation of *Campylobacter* spp. towards meropenem and of *C. jejuni* towards macrolides is still favorable.
- The agar diffusion test (ellipsoid test) is a cost-effective and easy-to-perform alternative to microdilution for testing meropenem. However, confirmation using microdilution should be done in cases of borderline results.
- The comparison of resistance data from *Campylobacter* isolates originating from German slaughter animals (published in the BVL’s Zoonoses Monitoring Report) and German patients yielded, as expected, very similar results, thus supporting the One Health concept.

## Notes

### Competing interests

The authors declare that they have no competing interests.

## Figures and Tables

**Table 1:**
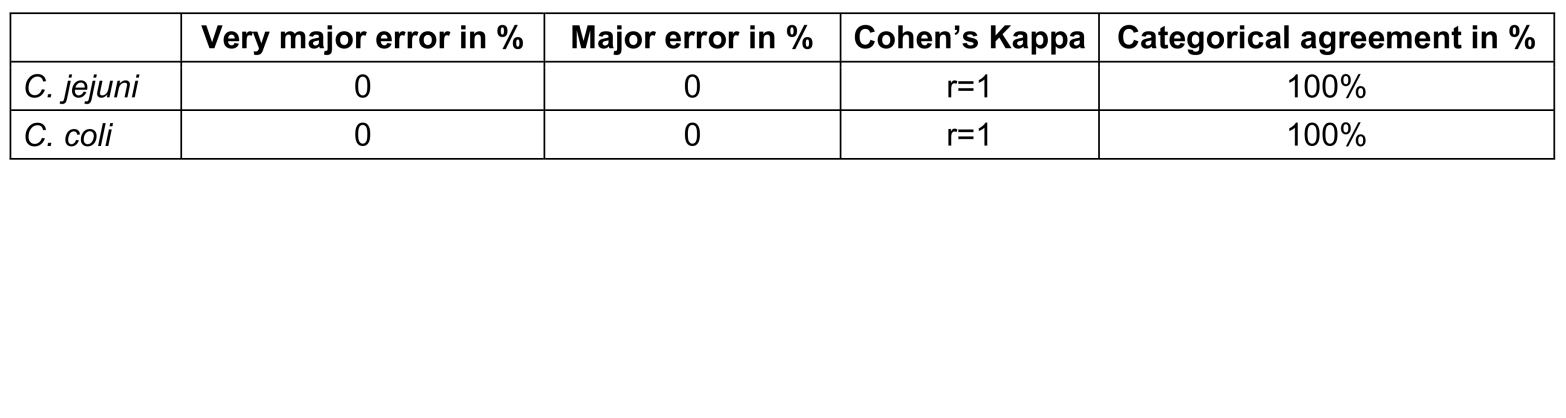
Major and very major errors, categorical agreement and Cohen’s Kappa for the interpretation as sensitive or resistant for the determination of meropenem MIC by ellipsoid test compared to broth microdilution of *Campylobacter jejuni* and *coli*

**Table 2:**
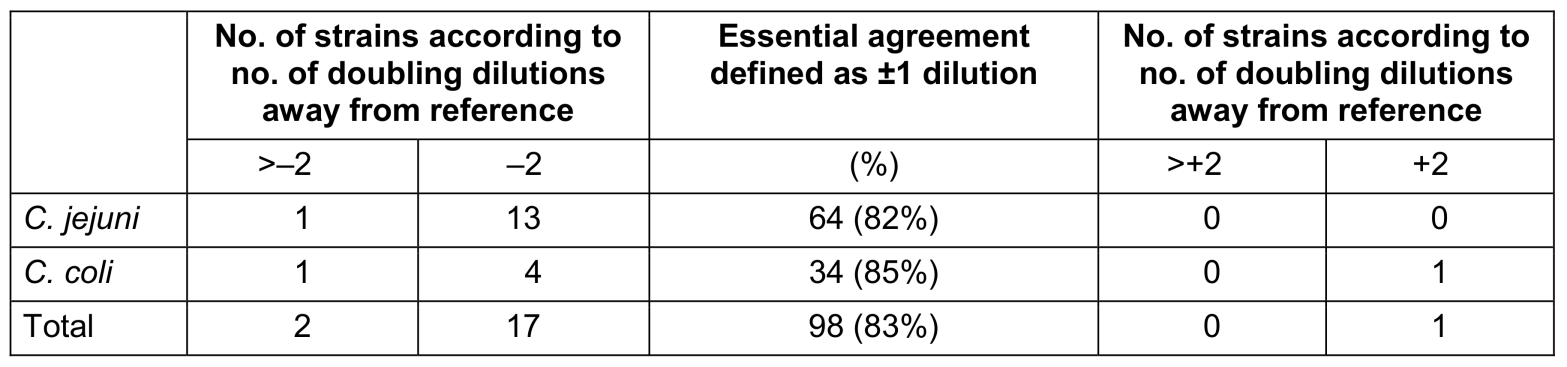
Essential agreement for ellipsoid test compared to broth microdilution (reference) for determination of meropenem MIC by ellipsoid test compared to broth microdilution of *Campylobacter* *jejuni* and *coli*

**Table 3:**
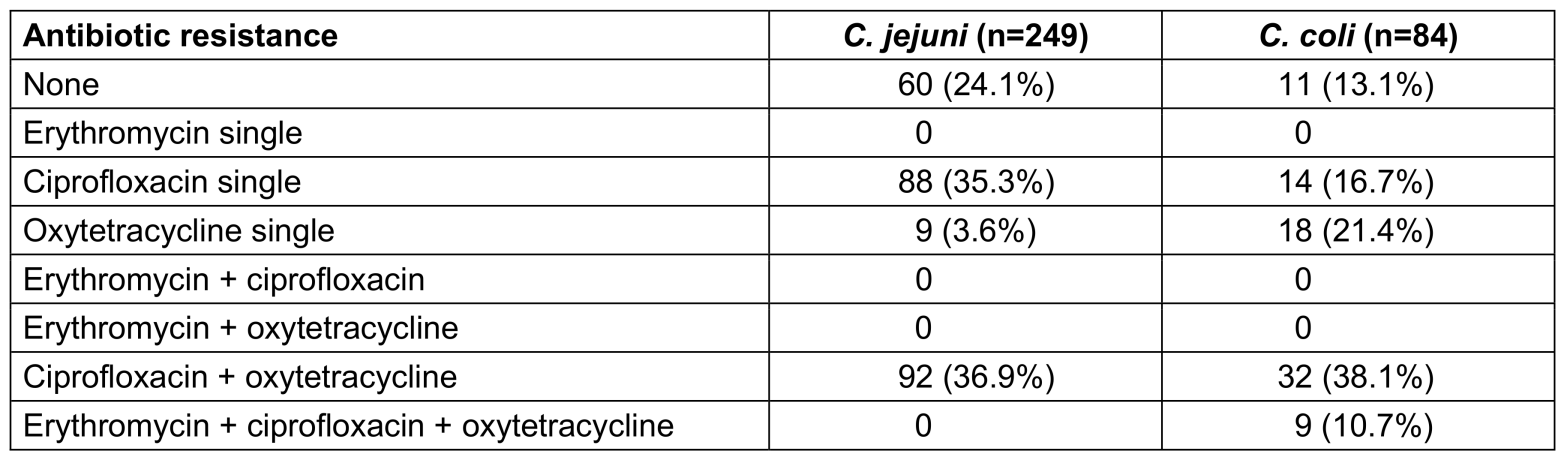
Distribution of single and combined resistance of *C. jejuni* and *C. coli* isolates to erythromycin, ciprofloxacin and oxytetracycline
